# Boric Acid Disturbs Cell Wall Synthesis in *Saccharomyces cerevisiae*

**DOI:** 10.1155/2010/930465

**Published:** 2010-12-27

**Authors:** Martin Schmidt, Jaron Z. Schaumberg, Courtney M. Steen, Michael P. Boyer

**Affiliations:** Biochemistry and Nutrition, Des Moines University, 3200 Grand Avenue, Des Moines, IA 50312, USA

## Abstract

Boric acid (BA) has broad antimicrobial activity that makes it a popular treatment for yeast vaginitis in complementary and alternative medicine. In the model yeast *S. cerevisiae*, BA disturbs the cytoskeleton at the bud neck and impairs the assembly of the septation apparatus. BA treatment causes cells to form irregular septa and leads to the synthesis of irregular cell wall protuberances that extend far into the cytoplasm. The thick, chitin-rich septa that are formed during BA exposure prevent separation of cells after abscission and cause the formation of cell chains and clumps. As a response to the BA insult, cells signal cell wall stress through the Slt2p pathway and increase chitin synthesis, presumably to repair cell wall damage.

## 1. Introduction

In the right amounts, boron is an essential nutrient for animals, plants, and fungi [1–3]. However, at high concentrations boric acid (BA) becomes an effective poison that is widely used for the killing of diverse organisms ranging from bacteria to rodents [4]. In medicine, BA is used as an alternative treatment for vaginal yeast infections [5]. While the molecular details of BA action on yeast remain unclear, it was recently shown that BA interferes with morphogenesis, to the effect that it inhibits the transition from the yeast to the hyphal form of the pathogenic yeast *C. albicans *[6]. Because the ability to switch to hyphal growth is an important virulence factor in *C. albicans *[7], suppression of such elongated growth by BA may in part explain its therapeutic effect. The present study was undertaken to assess the effect of BA on morphogenesis and cell wall synthesis in yeast, using the well-established model organism *Saccharomyces cerevisiae *as a study subject. 

In *S. cerevisiae*, morphogenesis and cell wall synthesis depend on the correct assembly of cytoskeletal proteins. To guide cell wall synthesis during cytokinesis, a ring of septin filaments forms during the G1 phase of the cell cycle and is subsequently completed into a contractile actomyosin ring (CAR) by the addition of myosin and actin, among other proteins [8, 9]. To complete abscission, the last phase of cytokinesis, the cells first separate mother and daughter cells with a chitin primary septum. The deposition of glucan and mannoprotein-rich cell wall material on the mother and daughter side of the primary septum later completes the trilaminar septum that can be observed under normal culture conditions. A disturbance in the assembly of the septation apparatus—the cohesive functional unit that constructs the primary septum—leads to the formation of highly aberrant septa [10, 11]. The septa formed under these conditions do not allow for the separation of cells after cytokinesis, leading to the formation of chains and clumps of misshaped cells. Based on the observation that BA causes such clumping and chain formation in *S. cerevisiae, *the present study was initiated to assess the influence of BA on the function of the septation apparatus.

## 2. Methods

### 2.1. Strains and Culture Conditions

Growth media and culture conditions were as described in [12]. Strains are listed in Table 1. 

Strain YMS415 (*CHS3::HA-HIS3*) was constructed by the short flanking homology method [15]. The *CHS3::3HA-HIS3 *fragment was amplified by PCR from plasmid pFA6a-3HA-His3MX6 with primers 5′-TATTCTCAATCGGAAGGAGGAAAGTGACTCCTTCGTTGCAGGTGGAGGTGGAGGTGGAGGTGGACGGATCCCCGGGTTAATTAA-3′ and 5′-TCAACTTGTAAGTATCACAGTAAAAATATTTTCATACTGTGAATTCGAGCTCGTTTAAAC-3′. Integration of the fragment was verified by PCR and western blotting. Transformants showed a wild-type like distribution of chitin in calcofluor white stained preparations, demonstrating full functionality of the Chs3p-HA fusion protein.

### 2.2. Determination of BA Sensitivity

A visual representation of BA sensitivity was obtained by serially diluting an overnight culture of yeast grown in YPD and spotting 5 *μ*L of cell dilutions on YPD plates with the indicated concentrations of BA. Growth was analyzed after 3-day incubation at 30°C. Minimal inhibitory concentrations were determined according to CLSI standards with the broth microdilution assay [16].

### 2.3. Staining Procedures and Fluorescence Microscopy

Viability staining of yeast cultures was performed by incubating cells in 0.2 mg/mL methylene blue in 50 mM KH_2_PO_4_ for 5 minutes at RT.

In order to visualize the distribution of chitin and glucan in the cell wall, cells were washed and incubated for 5 minutes in 0.01% calcofluor white or 0.5% aniline blue, respectively [17]. Fluorescence was observed with a standard diamidino-2-phenylindol (DAPI) filter set (Zeiss).

Filamentous actin was visualized by Alexa 568-phalloidin staining of yeast cells. Fifty mL yeast cultures at a titer of 1–5∗10^6^ cells/mL in YPD were fixed by the addition of formaldehyde to a final concentration of 4% and incubated for 10 minutes at room temperature. Cells were pelleted and incubated for 1 hour in phosphate buffered saline (PBS; 137 mM NaCl, 2.7 mM KCl, 10 mM Na_2_HPO_4_, 1.76 mM KH_2_PO_4_, pH 7.4) with 4% formaldehyde. After 2 washes in PBS, cells were suspended in 100 *μ*L PBS with 10 *μ*L of an Alexa 568-phalloidin solution (6.6 *μ*M in methanol; Invitrogen) and 1 *μ*L of 1 mg/mL calcofluor white. Cells were incubated for 1 hour in the dark and washed twice with PBS. Cells were then pelleted and taken up in 50 *μ*L ProLong Antifade reagent (Invitrogen) before mounting on slides.

GFP-tagged Cdc3p and Myo1p were observed with the GFP-filter set (Zeiss) in cells transformed with pRS316CDC3GFP [12] and pMS55 [11].

### 2.4. Electron Microscopy

Electron microscopic examination of yeast cell walls followed a previously published protocol [18].

### 2.5. Western Blotting

Analysis of Slt2p phosphorylation and total Slt2p by western blotting followed established protocols [19, 20]. As a loading control, an antibody directed against Tub4p (goat *χ*-tubulin yK18, Santa Cruz Biotechnology) was used at 1/1,000 dilution. For the determination of Chs3p-HA, cell membranes were isolated according to Orlean [21]. One volume of mercaptoethanol-free 2x sample buffer with 2% SDS (Bio-Rad) was added to the protein extracts and samples were loaded on 6% SDS polyacrylamide gels without boiling. Incubations with antibodies were performed as described above [18]. Antibodies used were a 1/5,000 dilution of anti-HA/1/5,000 dilution of goat antimouse HRP (Santa Cruz Biotechnology). Four independent experiments were performed and representative results are shown. Membranes were stained in 0.02% Coomassie blue R250 in 40% Methanol, 5% acetic acid to serve as loading controls.

### 2.6. Enzyme Measurements

For the determination of enzyme activities, cell membranes were isolated as previously described [21]. 

Determination of glucan synthase activity followed a protocol by Mol and coworkers [22]. A 40 *μ*L glucan synthase assay contained 20 *μ*L of membrane suspension at a protein concentration of 1 mg mL^−1^, 5 mM ^14^C-UDP-glucose (activity 1 × 10^9^ cpm mmol^−1^), 75 mM Tris-Cl pH 7.5, 25 mM KF, and—when indicated—with 20 *μ*M GTP-*γ*-S for the determination of maximal GS activity. The reaction was incubated for 1 hour at 30°C and then stopped by adding 1 mL 10% trichloroacetic acid (TCA). Reaction mixtures were filtered through a type A/E glass fiber filter (Pall). Filters were washed twice with 1 mL 10% TCA and four times with 70% ethanol. Filters were dried and taken up in 10 mL cytoscint ES scintillation fluid (ICN), and activity was recorded in a scintillation counter. Activities were calculated as c.p.m. incorporated h^−1^ *μ*g protein^−1^. 

**Figure 1:**
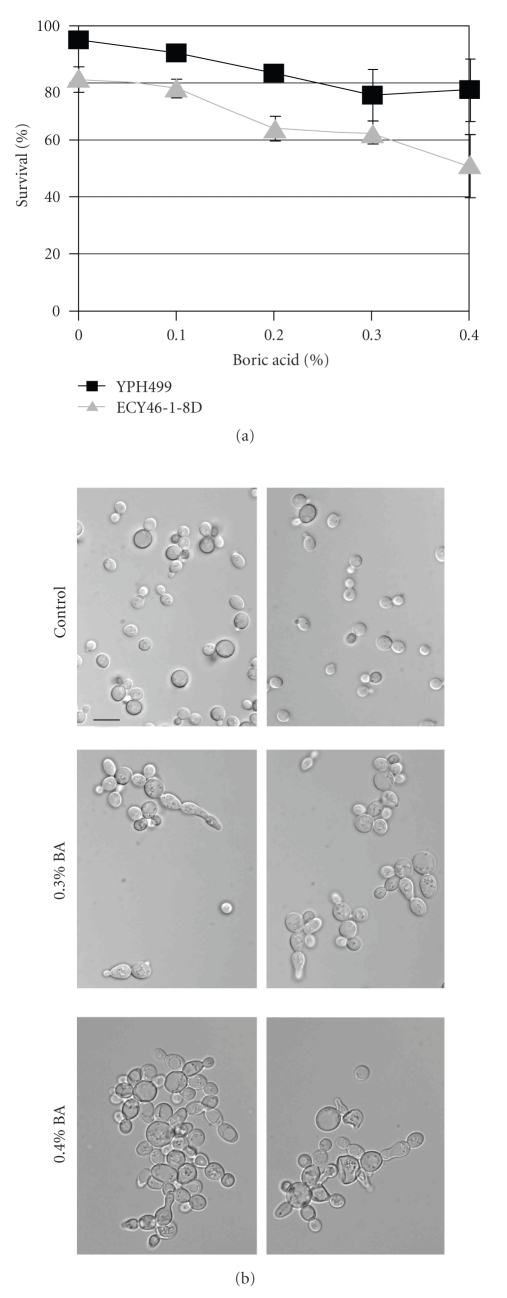
(a) Viability of strains YPH499 and ECY46-1-8D in the concentration range of 0–0.4% BA as determined by methylene blue staining. (b) Changes in morphology of YPH499 during exposure to BA. Cultures grown with BA show clumps and chains of incompletely separated cells. The scale bar represents 5 *μ*m.

Chitin synthesis was determined as described by Choi and Cabib [23]. To measure chitin synthase 2 and 3 activities, experiments were performed in the *chs1 *deletion strain ECY46-1-8D. The *chs1 *deletion was found to have no influence on BA sensitivity (Figure 1(a)). The chitin synthase assay mixture contained 20 *μ*L of membrane suspension at 1 mg protein mL^−1^, 5 *μ*L of 0.5 M Tris/Cl pH 7.8, 5 *μ*L of 20 mM cobalt acetate, 5 *μ*L of 10 mM ^14^C-UDP-GlcNAc (5000 c.p.m. *μ*L^−1^), and 2 *μ*L of trypsin solutions (Sigma) at concentrations from 0.25 to 2.0 mg mL^−1^ in a total volume of 46 *μ*L. For determination of chitin synthase 3 activity, 5 *μ*L of water were substituted with 5 *μ*L of 50 mM nickel acetate. Proteolysis was stopped after incubating for 15 minutes at 30°C by adding 2 *μ*L of a soybean trypsin inhibitor solution at 1.5x the concentration of the trypsin solution. Chitin synthesis was initiated by adding 2 *μ*L of 0.8 M GlcNAc. After incubating for 60 minutes at 30°C, chitin synthesis was stopped by adding 1 mL 10% TCA. Reaction mixtures were filtered, washed, and assayed in cytoscint fluid as described above. Assays containing cobalt only show activities of chitin synthases 2 and 3 while cobalt/nickel assays show the activity of chitin synthase 3. Chitin synthase 2 activity was calculated from the difference of both assays. Two independent experiments were conducted for chitin and glucan synthesis.

## 3. Results

### 3.1. BA Acts as a Fungistatic Agent That Inhibits Cytokinesis

Vital staining of strains YPH499 and ECY46-1-8D with 0.05% methylene blue showed that BA concentrations between 0.1 and 0.4% do not severely reduce cell viability (Figure 1(a)). In the examined range around the minimal inhibitory concentration of 0.31%, BA thus functions as a fungistatic agent that slows down proliferation but does not kill cells. Note that the decline in viability in *chs1 *deletion strain ECY46-1-8D parallels the decline in wildtype viability. Due to the previously reported lysis of daughter cells in *chs1 *mutants [24], strain ECY46-1-8D shows a higher fraction of dead cells in all of the examined samples. 

Clumping and chain formation of cells occurs at BA concentrations above 0.2%, with the most striking effect observed at 0.4% (Figure 1(b)). This clumping is the hallmark of a cytokinesis defect that causes daughter cells to remain attached to mother cells. A spot test of BY4742 cells on YPD plates showed that no growth occurs at BA concentrations above 0.5%—although viable cells can be retrieved even after 10 days of incubation under these conditions (data not shown).

### 3.2. BA Exposure Leads to Abnormal Deposition of Chitin and Glucan

**Figure 2:**
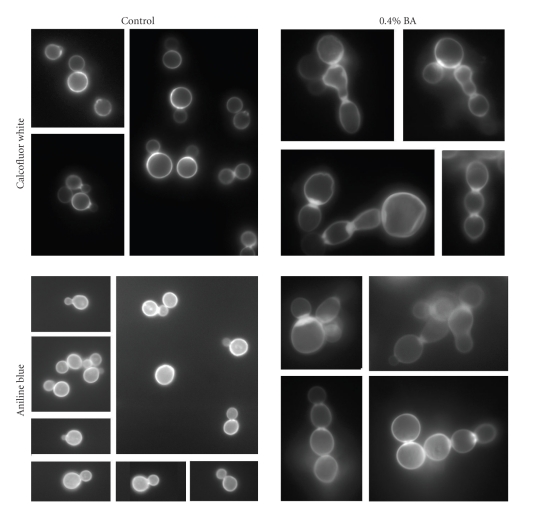
Analysis of chitin and glucan distribution in BA-treated cells: fluorescence of calcofluor white (top) and aniline blue (bottom) stained cells. At BA concentrations of 0.4%, chitin-rich—and to a lesser extent, glucan-rich—material accumulates at the bud neck. The thickening of septa is evident particularly between cells in a chain.

Staining of chitin and glucan in walls of cells grown with 0.4% BA revealed a buildup of cell wall material at bud necks, particularly in cell chains (Figure 2).

### 3.3. BA Exposure Interferes with the Localization of the Septation Apparatus

**Figure 3:**
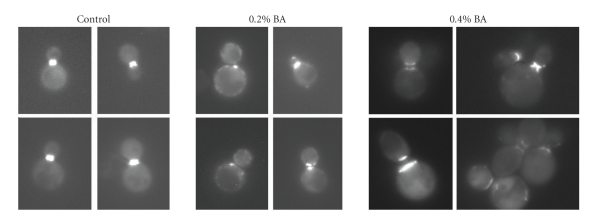
Appearance of septin (Cdc3-GFP) rings in BA-treated cells. Starting at a BA concentration of 0.2% and worsening at 0.4%, septin rings show irregular thickness and wide spacing between the mother and the daughter-side rings. BA also induces ectopic localization of septin patches (arrowheads).

In order to characterize the cytokinesis defect in BA-treated cells, the localization of key morphogenetic proteins at the bud neck was examined by fluorescence microscopy. It was found that BA influences the localization of the septin Cdc3, the cytokinetic myosin Myo1p, and the ring of filamentous actin that form sequentially in preparation for cytokinesis. Increasing concentrations of BA leads to the formation of Cdc3GFP rings that are disorganized, uneven, and not centered at the bud neck. Moreover, BA causes the formation of Cdc3GFP patches at sites other than the buck neck (Figure 3). 

**Figure 4:**
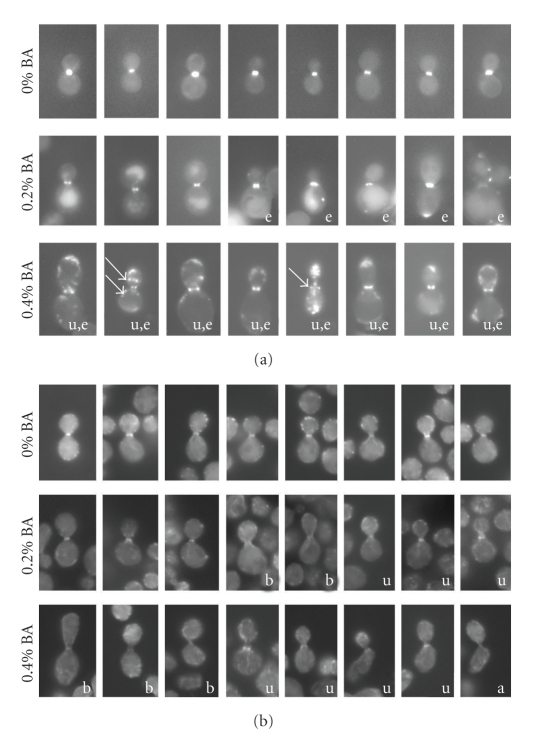
Appearance of the actomyosin rings in BA-treated cells. (a) Localization of Myo1GFP. Increasing concentrations of BA leads to irregular localization of Myo1GFP. Images show samples of normal, uneven rings not centered at the bud neck (u) and ectopic localization (e) of Myo1GFP patches. Note that at 0.4% BA Myo1GFP forms rings at locations other than the bud site that appear as bands in the selected images (arrows). (b) Localization of actin. The selection of images shows that increasing concentrations of BA causes actin rings to appear blurred (b) and uneven/not centered at the bud neck (u).

Imaging of Myo1GFP and actin shows that the formation of the CAR is impaired in a manner similar to the disturbance in septin ring organization. Increasing BA concentrations lowers the fraction of cells with Myo1GFP rings at the bud neck from 49 ± 5% at 0% to 25 ± 11% at 0.2% to 11 ± 11% at 0.4%. In addition, Myo1GFP rings formed under the influence of BA are often irregular in shape and at high concentrations (0.4%) ectopic localization of Myo1GFP patches at sites other than the bud neck is common (Figure 4(a)).

BA also impairs the assembly of the actin ring at the bud neck, which is the last component to be incorporated into the CAR before contraction starts (Figure 4(b)). Actin rings formed under the influence of BA become blurry, faint, and irregular in thickness. Under these conditions, it can be observed that some actin rings fail to localize to the narrowest point of the bud neck. In some cases, BA-treated cells even attempt cytokinesis without a detectable actin ring. Due to the low abundance of cells with actin rings in culture (< 2% in controls), quantification of rings yielded no statistically significant data.

### 3.4. BA Causes Ultrastructural Abnormalities in the Cell Wall

**Figure 5:**
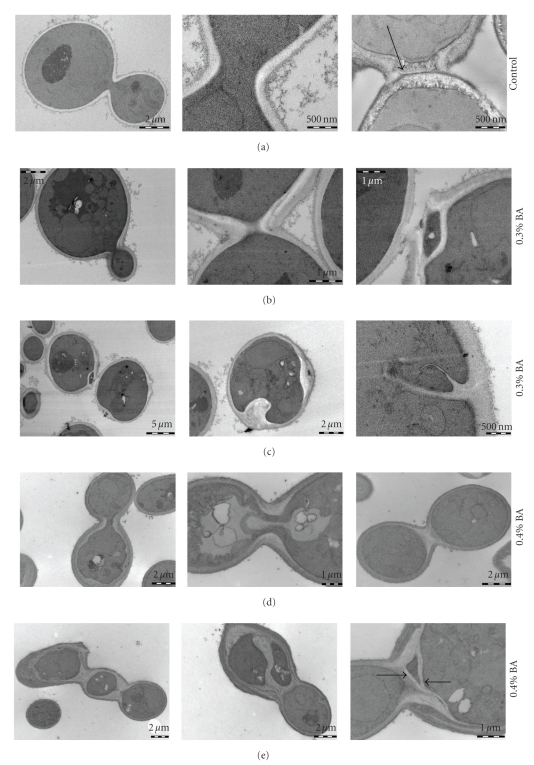
Electron micrographs of YPH499 cells grown in the presence of 0.3% and 0.4% BA. Cell wall chitin appears electron translucent. (a) No BA. Left: a mother-daughter pair; Middle: a close-up of the bud neck; Right: a completed trilaminar septum. Note the thin layer of chitin (primary septum; arrow) in the center of the structure. (b) Cells grown with 0.3% BA. Left: a mother-daughter pair; Middle: a close-up of the bud neck; Right: a completed septum. (c) Examples of other cell wall abnormalities in cells grown with 0.3% BA. Left: cell wall thickening and lacunae at the cell periphery; Middle: massive cell wall protuberances; Right: a septum forming at an ectopic location. (d) Cells grown with 0.4% BA. Left: a mother-daughter pair; Middle: a close-up of the bud neck; Right: a completed default septum. (e) Examples of other cell wall abnormalities in cells grown with 0.4% BA. Left: a chain of cells connected by default septa; Middle: a large cell wall structure spanning the entire length of the cell; Right: chitin-rich cell wall material deposited at the bud neck with two embedded aberrant septa (arrows).

Cultures of cells grown with 0.3% and 0.4% BA were examined by electron microscopy using a protocol that allowed for the visualization of chitin (Figure 5). The analysis of BA-treated cells showed massive abnormalities in the cell wall, particularly in the area of the septum, worse at 0.4% than at 0.3%. At these concentrations, BA prevented the construction of a single, straight chitin septum to separate mother and daughter cells. Instead, the cells synthesized protuberances extending far into the cytosol and large irregular septa, often at ectopic locations at the cell periphery.

### 3.5. Function of Slt2 in BA-Stressed Cells

**Figure 6:**
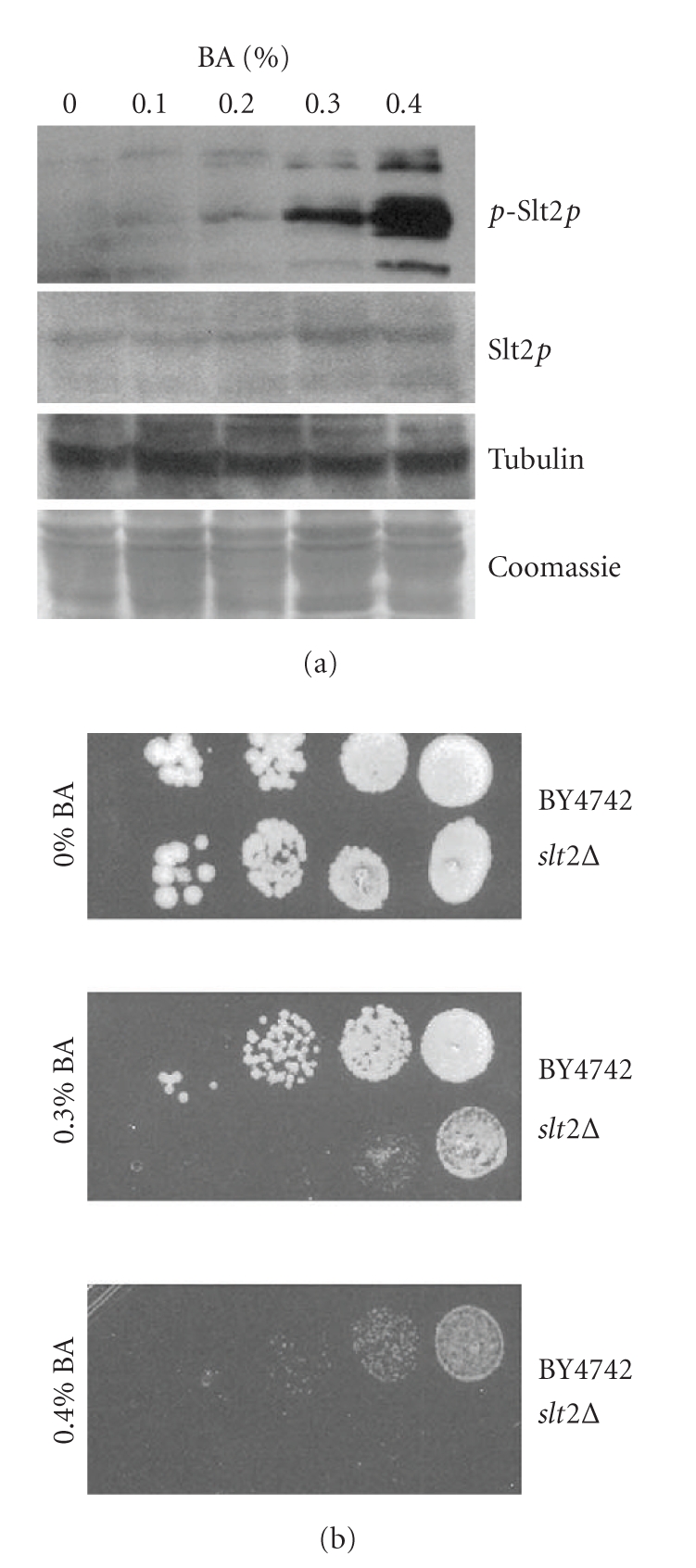
Slt2p-signaling in BA-treated cells. (a) Detection of phosphorylated Slt2p by western blotting in strain YPH499. Phosphorylation of Slt2p increases with escalating concentrations of BA. The total amount of Slt2p increases only slightly under the same condition. Gamma tubulin (Tub4p) detection by western blotting and the Coomassie blue-stained membrane are shown as loading controls. (b) BA sensitivity of an *slt2 *mutant strain compared to its WT BY4742 as determined by a spot assay.

In order to assess BA-induced cell wall integrity signaling, the amounts and the phosphorylation status of the signaling kinase Slt2p were determined by western blotting (Figure 6(a)). Phosphorylation of Slt2p increases with escalating concentrations of BA, suggesting that BA induces a cell wall integrity stress response. The dramatic increase in phosphorylation of Slt2p is accompanied by a weak increase in protein expression. Furthermore, it was determined that a *slt2* deletion mutant is sensitive to BA (Figure 6(b)), suggesting that Slt2p-signaling improves BA resistance by causing a transcriptional response to BA stress.

### 3.6. BA Stimulates Chitin Synthesis

**Figure 7:**
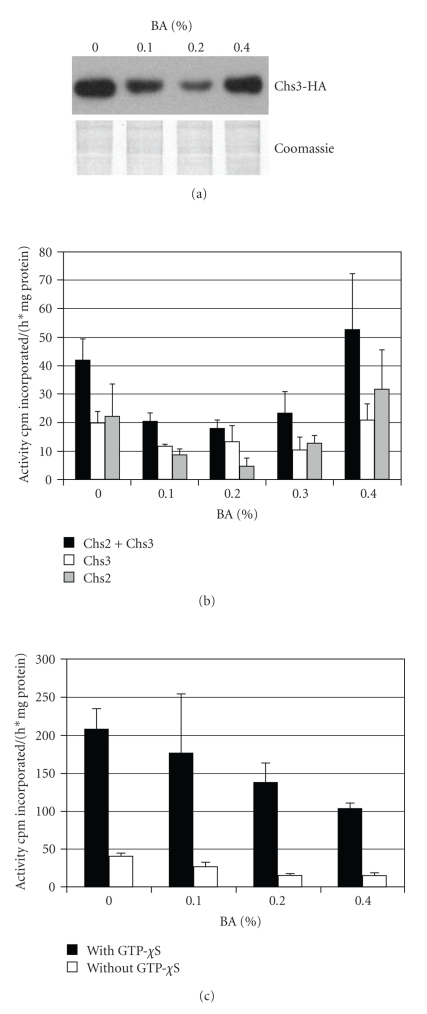
Glucan and chitin synthase activities in BA-treated cells. (a) Abundance of HA-tagged Chs3p in strain YMS415 treated with BA. Note the increase of Chs3p-HA at 0.4% BA. (b) Chitin synthase 2 and 3 activities in strain ECY46-1-8D. After a decline at lower BA concentrations, chitin synthase activities increase at 0.4% (c) Glucan synthase activities. Solid bars indicate activity after activation by GTP-*χ*S. Both maximal and physiological glucan synthase activities decline with increasing BA concentrations.

Western blotting showed a nonlinear correlation between BA concentration and abundance of Chs3p-HA (Figure 7(a)). While at BA concentrations of 0.1 and 0.2% the amount of Chs3p-HA decreases, there is an increase of Chs3p-HA at 0.4% BA. The increase of Chs3p-HA parallels the increase of chitin-rich septa visible in calcofluor white-stained cultures. 

The multiple posttranslational and protein targeting processes involved in Chs3p and Chs2p activation [9] necessitate enzymatic determination of chitin synthase activities in addition to detection of protein amounts.  Figure 7(b) shows that BA-induced changes in chitin synthase activities mirror the changes in Chs3p-HA amount (Figure 7(a)) and bud neck thickness (Figure 2). While at 0.1% and 0.2% BA decreases chitin synthase activities, presence of 0.3% and 0.4% BA increases chitin synthesis. At 0.4% BA, activities of both chitin synthase 2 and 3 exceed activities in control cultures. 

A measurement of glucan synthase activity in the membranes of strain YPH499 showed a constant decline with increasing BA concentrations (Figure 7(b)). The addition of GTP-*χ*S activates the GTP binding protein Rho1p and maximizes Fks1p activity. Neither maximal nor physiological (without addition of GTP-*χ*S) glucan synthase activity showed an increase at high concentrations of BA.

## 4. Discussion

Living organisms are constantly exposed to boron, a mineral that is abundant in soil and water. Boron is a weak acid and, at physiological pH, is present mostly as boric acid (H_3_BO_3_; BA). For each organism, there is an optimal BA concentration. Too little BA causes symptoms of deficiency while too much BA has a poorly defined cytotoxic effect [25]. Particularly the toxic effects of boron overload have led to a variety of applications for BA and related compounds, ranging from pest control to the treatment of vaginal yeast infections.

Boron toxicity is not yet understood. Molecular and *in vitro *studies suggest that boron participates in both enzymatic and nonenzymatic processes. The biological function of BA might be due to its reactivity with *cis-*hydroxyl groups on carbohydrate molecules [26]. Examples for boron-dependent reactions include plant hormone-sensitive NADH oxidase activity [27], the crosslinking of plant cell wall carbohydrates [28], and the mineralization of bone [29]. The molecular effects of boron lead to developmental defects in animals, particularly in the formation of the skeleton [30, 31]. These teratogenic effects of boron might be caused by direct or indirect inhibition of histone deacetylase activity [32] and a shift in Hox gene expression [31]. 

However, none of the above observations about BA toxicity serve to explain its effect on yeast—an organism that does not undergo genomic imprinting or Hox-dependent development. The only published data about the BA effect on yeast come from a recent study of the yeast *C. albicans* that suggested that BA impairs oxidative metabolism [6]. While this observation in itself is interesting and deserves further study, the authors also show that BA directly or indirectly influences the morphology of *C. albicans.* The present study expands on this observation by showing that BA disturbs morphogenesis in the model yeast *Saccharomyces cerevisiae*, particularly during cytokinesis. 

The data presented here show that BA impairs the formation of the primary septum in *S. cerevisiae*—a defect that can be explained by incorrect assembly of the cytoskeleton at the bud neck. The molecular machinery that constructs the chitin cell wall between mother and daughter cells, the septation apparatus, consists of chitin synthase 2, and a contractile actomyosin ring (CAR). The septation apparatus is assembled sequentially from myosin, chitin synthase 2 and actin on a scaffold of septins at the bud neck [8, 9]. Should the assembly of a functional septation apparatus fail, the cell is unable to construct an orderly primary septum by chitin synthase 2 and is forced to divide by depositing large amounts of chitin-rich cell wall material at the bud neck. The chitin in these so-called default septa is provided by chitin synthase 3 [10, 33, 34]. Since these irregular septum structures are resistant to degradation by chitinase, cells remain connected after cytokinesis and form cell chains and clumps [35]. In *S. cerevisiae, *the localization of the septum is determined by the position of the septins. A septin assembly that erroneously localizes to a site other than the bud neck will direct the formation of a septum-like structure at the respective location [34]. 

Our data show that in *S. cerevisiae *increasing concentrations of BA leads to an irregular assembly of the septin scaffold, an inability to position the Myo1 ring, and a failure to correctly assemble an actin ring at the bud neck. The aberrant localization and irregular appearance of the septin Cdc3GFP in BA-treated cells is an important key to understanding the septation defect. We propose that BA causes problems with the assembly of the septin scaffold which later impair the localization and function of the CAR, resulting in the formation of highly irregular cell wall structures. 

Like other threats to the integrity of the cell wall, septation defects trigger cell wall integrity signaling through the protein kinase C (PKC) pathway. Under these conditions, cell wall integrity signaling leads to hyperphosphorylation of the PKC downstream effector Slt2p [36–38], accompanied with a much weaker increase in Slt2p amount [39]. This will ultimately lead to the activation of cell wall repair enzymes, most notably chitin synthase 3 [36, 40]. The present study shows that BA activates yeast cell wall integrity signaling pathways as evidenced by BA concentration-dependent phosphorylation of Slt2p. Presumably in response to cell wall integrity signaling, cells increase the activity of the cell wall repair enzyme chitin synthase 3 in a BA concentration-dependent manner. The somewhat unusual pattern of chitin synthase induction—a decline at low concentrations followed by an increase at higher doses—should be interpreted based on the impact boric acid on growth. We propose that at concentrations below the MIC where the impact on growth is measurable but weak, boric acid stress reduces cell wall synthesis activity along with other metabolic activities. Once boric acid stress exceeds a tolerable limit—at concentrations above the MIC—the cell responds forcefully by induction of stress survival mechanisms such as chitin synthase 3-mediated cell wall reinforcement. 

 It is worth noting that the activity of the chitin septum-forming chitin synthase 2 also increases during BA exposure. It is evident that the increase in chitin synthase 2 activity correlates well with the increased number and size of chitin septa in BA-treated cells. However, since the regulation of chitin synthase 2 activity is not well understood, we dare not hazard an explanation for this phenomenon. 

The data presented in this study show that in *S. cerevisiae *BA disturbs the localization of the contractile actomyosin ring secondary to causing irregularities in the septin scaffold at the bud neck. In agreement with the reviewed literature we propose that the aberrant localization of the septins ultimately impedes the formation of the primary septum, which leads to the synthesis of thick, chitin-rich default septa. In addition, the localization of septins at sites other than the bud neck explains the synthesis of cell wall protuberances that should be interpreted as incomplete ectopic septa [34]. Furthermore, our data show that a BA-induced septation defect, just like other septation defects, triggers cell wall integrity signaling through the Pkc1-Slt2 pathway and results in increased chitin synthase 3 activity.

## Figures and Tables

**Table 1:** Strains.

Strain	Genotype	Source
BY4742	*MAT*α* his3Δ1 leu2Δ 0 lys2Δ0 ura3Δ0*	Invitrogen

BY4742 *slt2*::*kanMX6 *	*MAT*α* his3Δ1 leu2Δ0 lys2Δ 0 ura3Δ 0 slt2::kanMX6*	Invitrogen

ECY46-1-8D	*MAT * **a ** *ura3-52 lys2-801 ade2-101 trp1-Δ63 his3-Δ200 leu2-Δ 1chs1::HIS3*	[13]

YMS415	*MAT * **a ** *ura3-52 lys2-801 ade2-101 trp1-Δ63 his3-Δ200 leu2-Δ1 CHS3::3HA-HIS3*	This study

YPH499	*MAT * **a ** *ura3-52 lys2-801 ade2-101 trp1-Δ63 his3-Δ200 leu2-Δ1*	[14]
